# Depletion of Resident Macrophages Does Not Alter Sensory Regeneration in the Avian Cochlea

**DOI:** 10.1371/journal.pone.0051574

**Published:** 2012-12-11

**Authors:** Mark E. Warchol, Reto A. Schwendener, Keiko Hirose

**Affiliations:** 1 Department of Otolaryngology, Washington University School of Medicine, St. Louis, Missouri, United States of America; 2 Department of Anatomy and Neurobiology, Washington University School of Medicine, St. Louis, Missouri, United States of America; 3 Institute of Molecular Cancer Research, University of Zurich, Zurich, Switzerland; University of Washington, Institute for Stem Cells and Regenerative Medicine, United States of America

## Abstract

Macrophages are the primary effector cells of the innate immune system and are also activated in response to tissue injury. The avian cochlea contains a population of resident macrophages, but the precise function of those cells is not known. The present study characterized the behavior of cochlear macrophages after aminoglycoside ototoxicity and also examined the possible role of macrophages in sensory regeneration. We found that the undamaged chick cochlea contains a large resting population of macrophages that reside in the hyaline cell region, immediately outside the abneural (inferior) border of the sensory epithelium. Following ototoxic injury, macrophages appear to migrate out of the hyaline cell region and towards the basilar membrane, congregating immediately below the lesioned sensory epithelium. In order to determine whether recruited macrophages contribute to the regeneration of sensory receptors, we quantified supporting cell proliferation and hair cell recovery after the elimination of most resident macrophages via application of liposomally-encapsulated clodronate. Examination of macrophage-depleted specimens at two days following ototoxic injury revealed no deficits in hair cell clearance, when compared to normal controls. In addition, we found that elimination of macrophages did not affect either regenerative proliferation of supporting cells or the production of replacement hair cells. However, we did find that macrophage-depleted cochleae contained reduced numbers of proliferative mesothelial cells below the basilar membrane. Our data suggest that macrophages are not required for normal debris clearance and regeneration, but that they may play a role in the maintenance of the basilar membrane.

## Introduction

Sensory transduction in the inner ear is mediated by hair cells, which detect sound vibrations and head movements, and provide synaptic input to afferents of the eighth cranial nerve. Hair cells – along with their accompanying supporting cells - reside in epithelial sheets that form barriers between the two fluid spaces of the inner ear (perilymph and endolymph). Cells within these sensory epithelia can be injured or killed by acoustic trauma, treatment with ototoxic drugs, or as a consequence of aging. In order to preserve the integrity of the inner ear fluid chambers, it is essential that cellular debris be quickly removed after injury or apoptosis. Epithelial debris clearance can be mediated by several distinct mechanisms. Cell corpses may be actively extruded from the epithelium [1], removed by resident or recruited macrophages, or engulfed by surrounding cells (acting as ‘amateur phagocytes’). In the avian cochlea, most dying hair cells appear to be extruded from the sensory epithelium [2], while recent evidence suggests that apoptotic vestibular hair cells are phagocytosed by adjacent supporting cells [3]. Although the sensory organs of the avian inner ear contain resident populations of macrophages [4], it is not clear whether those cells also participate in the removal of hair cell debris. The avian inner ear also has a robust ability to regenerate hair cells after injury, and prior studies have suggested that resident macrophages may help initiate this regenerative process [4], [5], [6].

In light of the uncertain role of macrophages in the inner ear, the aims of the present study were to determine whether macrophages are required for removal of hair cell debris after ototoxic injury and to test the hypothesis that resident macrophages promote hair cell regeneration. Experiments were conducted on organotypic cultures of the chick cochlea, which retains its regenerative ability when maintained *in vitro* [7], [8]. Liposomally-encapsulated clodronate was used to deplete resident macrophages in cochlear cultures, and the resulting effects on debris clearance and sensory regeneration were quantified. Although ototoxic injury caused increased numbers of macrophages to migrate towards the lesioned regions, we found that the removal of macrophages did not affect the removal of apoptotic hair cells from the sensory epithelium. Instead, dying hair cells were quickly extruded from the lumenal surface of the epithelium in both normal and macrophage-depleted cochleae. Regenerative proliferation of cochlear supporting cells was also unaffected by macrophage removal, although we did observe reduced proliferation among cells below the basilar membrane. Finally, we observed no differences in hair cell recovery in macrophage-depleted cochleae vs. cochleae with normal macrophage populations. Together, these findings suggest that macrophages are not essential for the clearance of apoptotic hair cells or for the initiation of sensory regeneration. However, our findings suggest that macrophages may participate in the maintenance of the basilar membrane.

## Methods

### Animals

Fertilized chicken eggs were obtained from Charles River (Franklin, CT) and incubated until hatching. Chicks were then housed in heated brooders for 10–15 days, prior to use in experiments. All protocols were approved by the Animal Studies Committee of Washington University.

### Ototoxic Injury in vivo

Cochlear hair cells were lesioned *in vivo* through systemic treatment with streptomycin sulfate. Streptomycin was dissolved in 0.9% NaCl and chicks were given intramuscular injections of 1,200 mg/kg. Injections were given at ∼12∶00 PM for three consecutive days. At 24 hr after the final injection, chicks were euthanized via CO_2_ asphyxiation and cochleae were removed, fixed 30 min in 4% paraformaldehyde (in 0.1 M phosphate buffer) and processed for immunohistochemistry.

### Preparation of Organotypic Cultures

Cultures of the cochlea (basilar papilla) were prepared following previously described methods [4], [8]. Chicks were euthanized via CO_2_ asphyxiation and decapitated. Following removal of the skin and mandible, heads were placed in 70% EtOH for 5–10 min., in order to kill surface pathogens. The temporal bones were opened and cochleae were quickly explanted and placed in chilled Medium 199 (M199) with Hank’s salts and HEPES buffer. Fine forceps were used to remove the tegmentum vasculosum from each cochlea, and the lagena was also cut away using iridectomy scissors. However, the tectorial membranes were not removed from any of the specimens, either before placement in organotypic culture or prior to immunohistochemical processing. Individual dissected cochleae were transferred into culture wells (MatTek) that contained 100 µl of M199 with Earle’s salts 2,200 mg/l sodium bicarbonate, 0.69 mM L-glutamine, 25 mM HEPES, supplemented with 1% FBS. Cochleae were initially incubated in medium that contained 1 mM streptomycin sulfate. After 24 hr of streptomycin treatment, all specimens were rinsed 3× in fresh medium and maintained for an additional 2–7 days in streptomycin-free medium. Cultured specimens were maintained at 37°C in a 5%CO_2_/95% air environment, and were fed fresh medium at two-day intervals.

### Clodronate Depletion of Macrophages

Liposomally-encapsulated clodronate (∼18 mg/ml clodronate [9]) was stored at −80°C and thawed immediately prior to use. Liposomes were then added to cochlear cultures at a dilution of 2 µl liposome solution to 100 µl medium, so that single cochleae were exposed to ∼36 µg clodronate. Control cultures were treated with equal volumes of ‘empty’ (PBS-containing) liposomes, or received no liposomal supplement. Specimens were incubated in these media for 24 hr and were then rinsed 3× over 30–60 min. and used for further experimentation.

### Identification of Proliferating Cells

Proliferating (S-phase) cells were labeled by the addition of bromodeoxyuridine (BrdU, 3 µg/ml) to culture medium. Specimens were incubated with BrdU for four days; BrdU-containing medium was replaced with fresh medium every 48 hr.

### Immunohistochemistry

All cultures were fixed for 30 min with 4% paraformaldehyde (PFA; in 0.1 M phosphate buffer). Specimens were then thoroughly rinsed with PBS and nonspecific antibody binding was blocked by incubation for 2 hr in PBS with 5% normal horse serum (NHS) and 0.2% Triton X-100. Hair cells were identified with an antibody against myosin VIIA (Proteus Biosciences, rabbit polyclonal, 1∶500). Macrophages were labeled with the KUL01 antibody (1∶100, mouse monoclonal, Southern Biotechnology or Santa Cruz Biotechnology [10]). Immunohistochemistry for BrdU was performed using previously published methods [11]. Specimens were maintained in primary antibodies overnight and were then rinsed 5× in PBS and incubated for 2 hr. in secondary antibodies (Alexa-488 donkey anti-mouse, Alexa-546 donkey anti-rabbit, 1∶500) and/or Alexa-488 or Alexa-546 phalloidin (all Life Technologies; Carlsbad, CA). The secondary antibody solutions also contained DAPI, in order to label cell nuclei. All immunolabeling was done at room temperature.

### Imaging and Data Analysis

**Figure 1:**
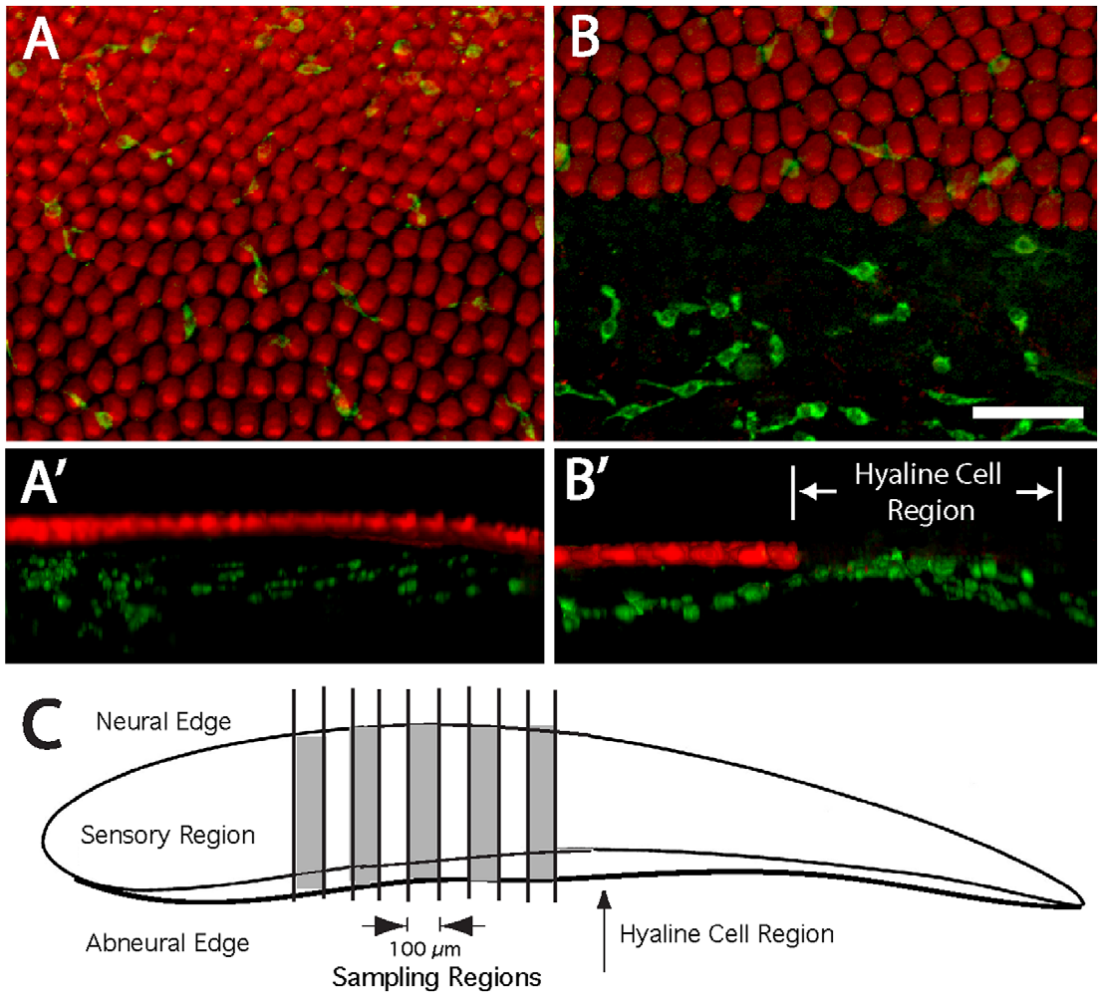
Macrophages in the uninjured chick cochlea. Surface views revealed macrophages (green) associated with the sensory region (A). In addition, numerous macrophages were present in the hyaline/cuboidal cell region, which is immediately outside the inferior border of the sensory epithelium (B). Three-dimensional reconstructions of confocal image stacks indicated that macrophages in the sensory region were mainly located below the sensory epithelium (A′, B′). Macrophages in the sensory and nonsensory (hyaline cell) regions of the cochlea were quantified from five 100 µm-wide strips, located in the midregion of the cochlea (shaded areas, C). Green: KUL01 (macrophages), Red: phalloidin. Scale bar = 30 µm.

Confocal microscopy (Zeiss LSM 700) was used for all imaging. Image stacks were reconstructed and processed using Volocity software (PerkinElmer) and stored as TIFF files. All quantification was conducted offline, from stored images. Macrophages within the sensory and hyaline/cuboidal cell regions were counted from 100 µm-wide strips, beginning ∼1 mm from the distal tip of the cochlea and proceeding toward the proximal end (see Fig. 1C). Surviving and regenerated hair cells were counted from five 100×100 µm regions/cochlea. These sampled regions were located approximately midway between the superior and inferior edges and the proximal and distal tips. A similar sample scheme was used to quantify BrdU-labeled cells. All data are presented as mean±SD, as computed by Microsoft Excel. Statistical significance was assessed using Students t-test (2-tailed, Microsoft Excel).

## Results

### Response of Cochlear Macrophages to Ototoxic Injury in vivo

In initial studies, we used the KUL01 antibody to characterize the distribution of macrophages in the normal (undamaged) chick cochlea. Surface views revealed moderate numbers of macrophages associated with the sensory region (Fig. 1A, B). However, three-dimensional reconstructions of confocal images indicated that almost all such macrophages were located below the sensory epithelium, near the basilar membrane (Fig. 1A′). We also observed numerous macrophages within the hyaline/cuboidal cell region, just outside of the inferior border of the sensory epithelium (e.g., Fig. 1B, B′). Macrophages in the sensory epithelium were quantified from five 100 µm-wide strips that extended from the neural to abneural border and were oriented perpendicular to the tonotopic axis. The sampled regions were located in the widest portion of the sensory epithelium (i.e., ∼1–2 mm from the distal tip) and were separated from each other by 100 µm. We also quantified the numbers of macrophages in the adjoining hyaline cell regions of these same sampled regions (see Fig. 1C). Uninjured cochleae contained 2.8±1.7 macrophages/100 µm (n = 25 samples from five specimens) within the sensory region, while the adjoining hyaline/cuboidal cell region contained 8.8±3.1 macrophages/100 µm. This observation indicates that the hyaline/cuboidal cell region (which is only ∼15% as wide as the sensory region) contains a relatively large proportion of resting macrophages.

**Figure 2:**
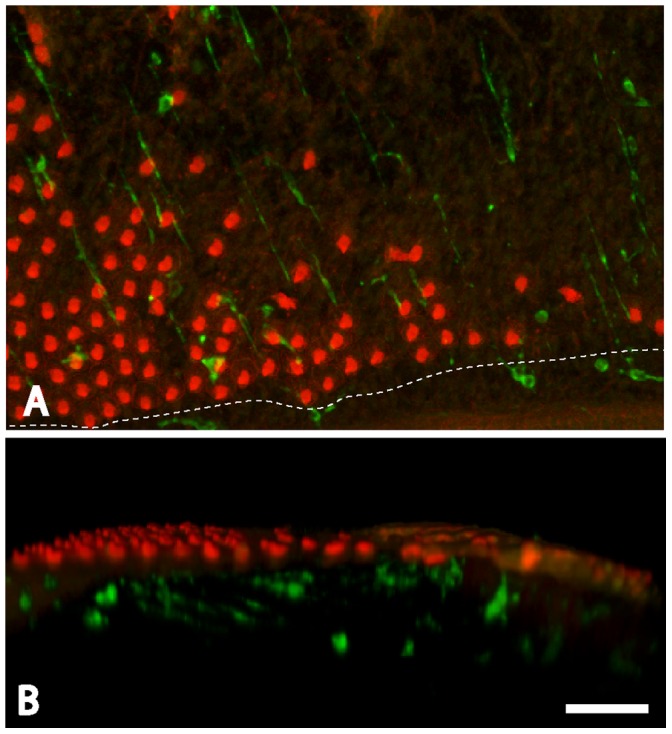
Response of cochlear macrophages to ototoxic injury. Chicks received three injections of streptomycin (1,200 mg/kg, 1/day), which killed most hair cells in the proximal-most ∼50% of the sensory region. Cochleae were fixed 24 hr after the final injection, and macrophages were identified by immunoreactivity for KUL01 (green). Hair cell stereocilia and cell-cell junctions were also labeled with phalloidin (red). Surface views (A) indicated that the sensory regions of lesioned cochlea contained increased numbers of macrophages. The dashed line indicates the inferior border of the sensory epithelium. Examination of three dimensional renderings of the confocal image stacks (B) indicated that most newly-recruited macrophages remained below the sensory epithelium. Scale bar = 30 µm.

**Figure 3:**
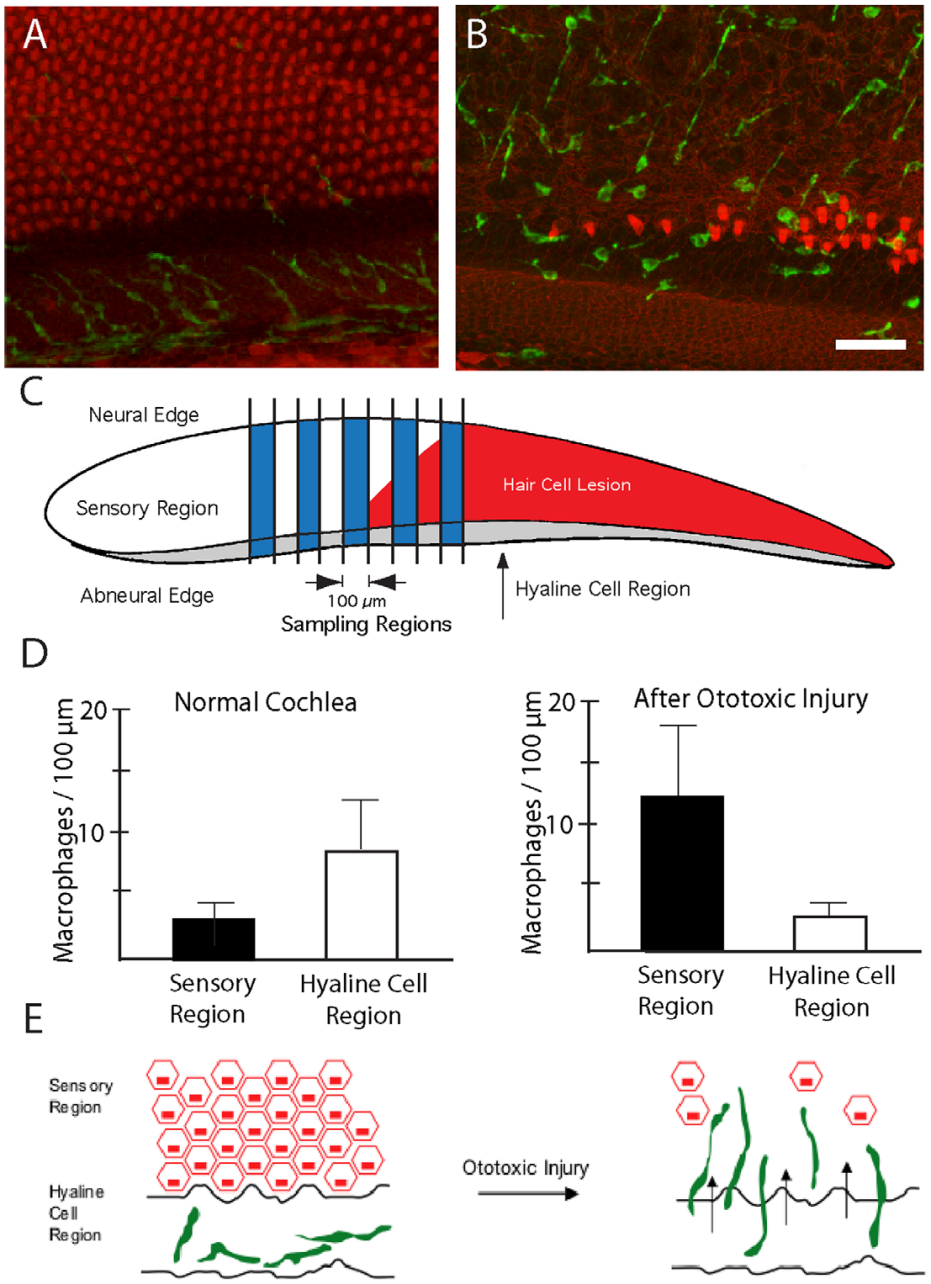
Redistribution of cochlear macrophages following ototoxic injury. The hyaline cell regions of undamaged cochleae contained numerous macrophages (A, green). After ototoxic injury, increased numbers of macrophages were observed within the sensory region (B). Macrophage numbers in the sensory and hyaline regions were quantified using the scheme shown in (C). Data from undamaged cochleae indicated that the hyaline cell region contained more macrophages than did the adjacent sensory region (D, *left*, p<0.001). After injury, however, macrophages in the sensory region outnumbered those in the hyaline region (D, *right*, p<0.001). This apparent redistribution suggests that resting macrophages migrate into the sensory region in response to hair cell injury, as schematized in (E). Scale bar = 30 µm.

**Figure 4:**
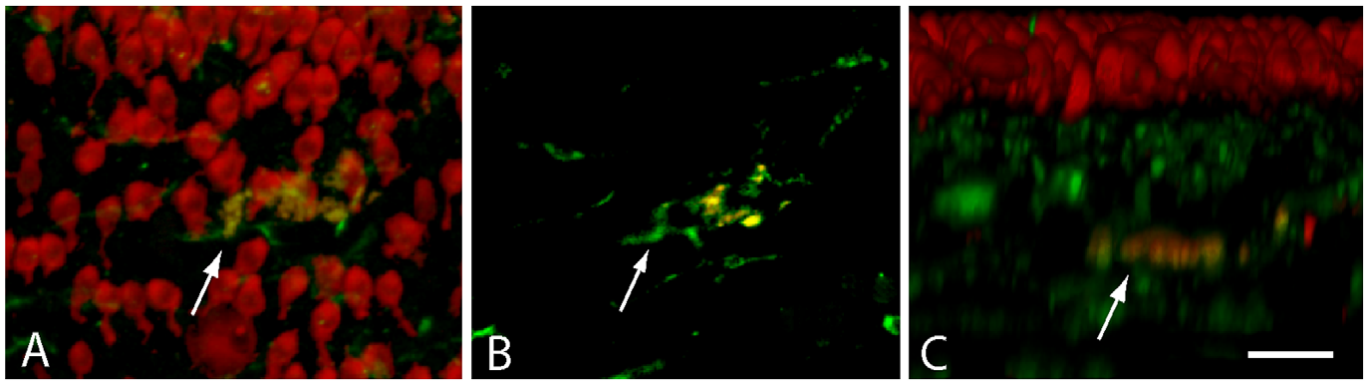
Co-localization of macrophages with hair cell debris. Cochleae were fixed at one day after completion of streptomycin injections (see text) and immunolabeled for KUL01 (green, macrophages) and myosin VIIA (red, hair cells). Specimens contained a small number of cells that were double-labeled for both markers (arrows). Such labeling is suggestive of macrophages having phagocytosed the remains of injured hair cells. Reconstruction of confocal image stacks revealed that such cells were below the sensory epithelium (C). Scale bar = 30 µm.

We next examined changes in the numbers and distribution of cochlear macrophages following aminoglycoside ototoxicity. Chicks were given three injections of streptomycin (1,200 mg/kg, 1/day, for three consecutive days), which killed nearly all hair cells in the proximal ∼50% of the sensory epithelium. Surface views of cochleae fixed one day after streptomycin injections revealed enhanced numbers of macrophages within the lesioned sensory regions (Fig. 2A, B). Notably, this increase appeared to be accompanied by a corresponding decrease in the numbers of macrophages in the hyaline/cuboidal cell region (Fig. 3A, B). In order to confirm a redistribution of macrophages after hair cell injury, we counted the numbers of macrophages/100 µm in both the sensory and the hyaline cell regions (Fig. 3C). At one day after completion of streptomycin injections, the sensory region contained 12.2±6.4 macrophages/100 µm, while the hyaline cell region contained 2.6±1.6 macrophages/100 µm (Fig. 3D; n = 25 samples from five specimens). This redistribution of macrophages suggests that, after ototoxic injury, macrophages migrate from the hyaline cell region into the sensory region (e.g., Fig. 3E). Most of the newly-recruited macrophages remained below the sensory epithelium, although occasional macrophages appeared to extend processes into the sensory region (Fig. 2B). Also, since one possible function of tissue macrophages is to remove apoptotic cells, we looked for evidence of phagocytosis of hair cell debris. Cochleae were fixed at one day after completion of streptomycin injections and were then immunolabeled for KUL01 (to identify macrophages) and myosin VIIA (to label the remnants of apoptotic hair cells). Occasional cells labeled with both antibodies were observed (Fig. 4), but such cells were rare (∼1–2/cochlea). This observation, combined with the fact that most macrophages remained well below the sensory epithelium, suggests that phagocytosis of apoptotic hair cells is not a primary function of cochlear macrophages.

### Selective Elimination of Macrophages from Organotypic Cultures of the Chick Cochlea

**Figure 5:**
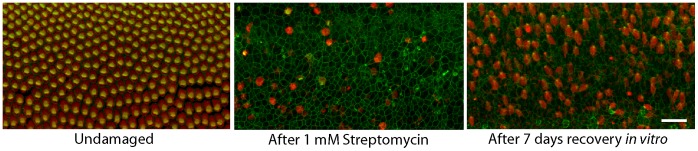
Ototoxic injury and hair cell regeneration in organotypic culture. Cultures of chick cochleae were treated for 24 hr with 1 mM streptomycin, which killed nearly all hair cells in the sensory epithelium (center). When such lesioned specimens were maintained for an additional seven days in streptomycin-free medium, significant hair cell recovery was observed (right). All images show the midregion of the chick cochlea. Labels: red-Myosin VIIA (hair cells), green-phalloidin. Scale bar = 30 µm.

**Figure 6:**
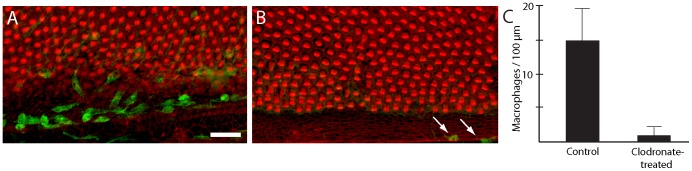
Treatment with liposomally-encapsulated clodronate reduces the number of macrophages in cochlear cultures. (A) Untreated cochlear cultures possess numerous macrophages (green), particularly in the hyaline/cuboidal cell region. (B) After treatment for 24 hr in clodronate-containing liposomes, very few macrophages remain (arrows). In contrast, hair cell morphology did not appear to be affected by clodronate treatment (red: phalloidin). (C) Quantification of macrophages in clodronate-treated vs. control cultures (n = four samples/specimen from eight clodronate-treated and six control cochleae) demonstrates a sharp reduction in the resident macrophage population after clodronate treatment (p<0.001). Scale bar = 30 µm.

The processes of hair cell injury, macrophage recruitment, and hair cell regeneration can be readily observed in organotypic cultures of the chick cochlea (Fig. 5) [4], [7], [8], [12]. In order to assess the role of macrophages in regeneration, we first examined whether macrophages can be selectively eliminated from cochlear cultures. Studies of macrophage influences in other somatic tissues (conducted both *in vivo* and *in vitro*) have employed liposomally-encapsulated clodronate to reduce macrophage numbers [9], [13]. Clodronate is a bisphosphonate compound that is toxic to macrophages when internalized (i.e., though the phagocytosis of clodronate-containing liposomes). Cultured cochleae (n = 8) were treated for 24 hr in medium that contained clodronate liposomes. Specimens were then thoroughly rinsed and maintained in culture for an additional 24 hr in clodronate-free medium. Control cultures (n = 6) were run in parallel, but did not receive clodronate liposomes. Following fixation, specimens were processed for KUL01 immunohistochemistry and macrophages were quantified from 100 µm segments in the cochlear mid-region (e.g., Fig. 1C, with sensory and hyaline cell region macrophages combined together). In all cases, we found that treatment with clodronate liposomes caused a dramatic reduction in the numbers of macrophages (Fig. 6). Significantly, we observed no evidence for hair cell damage in the clodronate-treated cultures. The inferior regions of clodronate-treated cochleae contained 73.7±10.5 hair cells/10,000 µm^2^, while comparable regions of control cochleae contained 74.0±8.2 hair cells/10,000 µm^2^ (n = 4 samples from each specimen). These observations suggest clodronate can eliminate macrophages from the chick cochlea without affecting hair cell survival.

### Macrophages are not Required for the Clearance of Apoptotic Hair Cells

**Figure 7:**
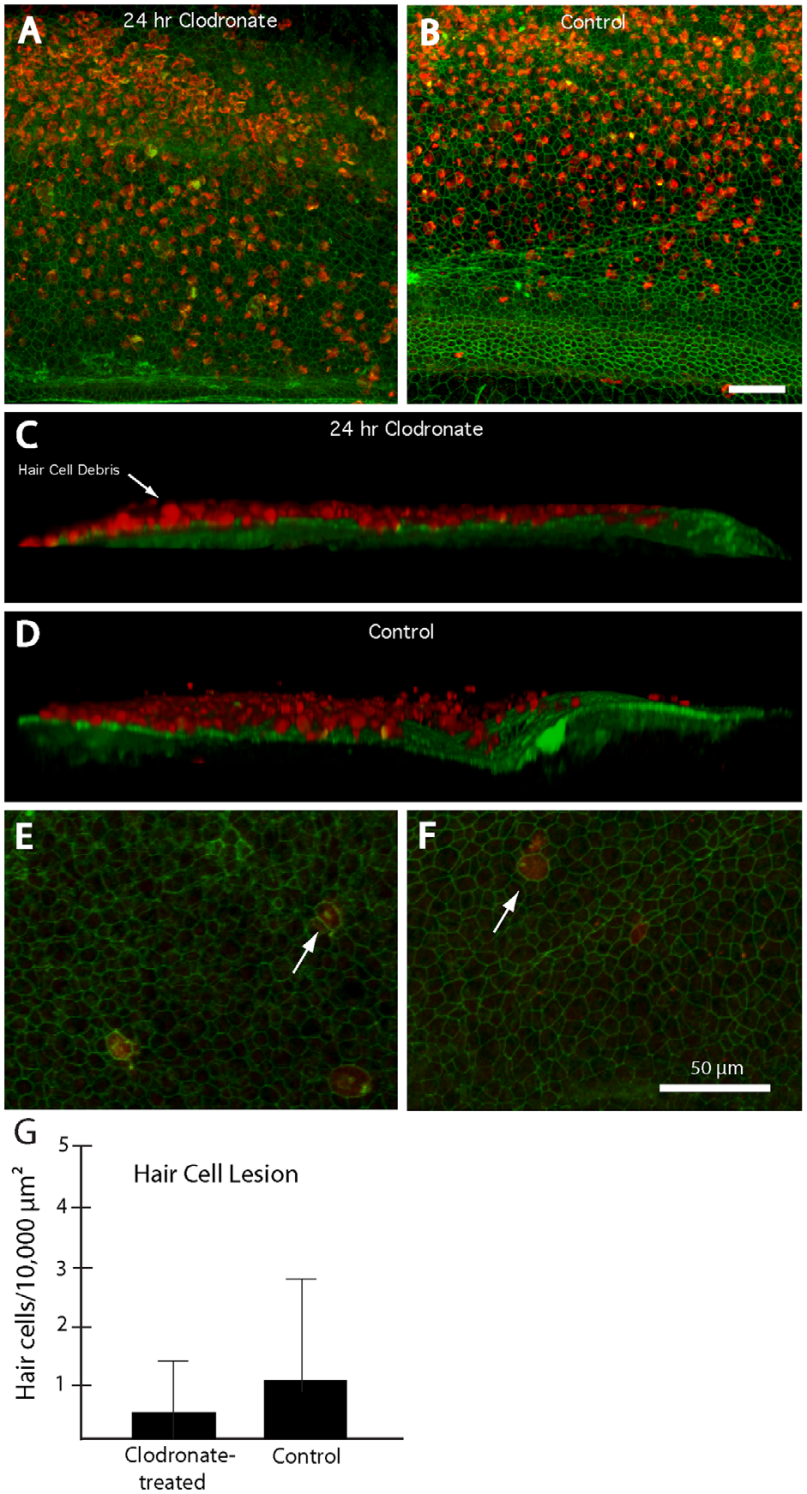
Elimination of macrophages does not affect the clearance of hair cell debris after ototoxic injury. (A, B) Surface views of cochleae fixed at 48 hr after streptomycin treatment. High levels of hair cell debris (labels: red-myosin VIIA; green-phalloidin) were present in all specimens, regardless of pretreatment. (C, D) Three-dimensional reconstructions of confocal image stacks indicated that nearly all hair cell debris was located above the lumenal surface of the sensory epithelium. (E, F) In contrast, confocal images that were confined to the sensory region revealed very low levels of hair cell debris remaining in the epithelia of either clodronate-treated (E) or control (F) cultures. Arrows in (E) and (F) point to myosin VIIA-labeled cells that remained within the sensory epithelium at 48 hours after ototoxic injury. Quantification of such cells confirms that macrophage depletion did not impair the removal of hair cell debris from the sensory epithelium (G). Scale bars = 50 µm.

In most tissues, a primary function of resident macrophages is the removal of cellular debris after apoptosis. We predicted that, if cochlear macrophages were essential for clearing dying hair cells after ototoxic injury, then the sensory epithelia of cochleae that lacked resident macrophages should contain numerous fragments of hair cell debris. To test this prediction, cochleae (n = 20) were placed in organotypic culture; ten specimens received liposomal clodronate while the remaining cochleae served as untreated controls. After 24 hours, all specimens were rinsed 3× and then treated for 24 hr in 1 mM streptomycin, in order to kill hair cells. Following ototoxic injury, the specimens were thoroughly rinsed and maintained *in vitro* for an additional 48 hr (to allow hair cells to complete the apoptotic process). Specimens were then fixed and labeled with phalloidin and immunolabeled for myosin VIIA. We observed widespread myosin VIIA immunoreactivity in all cochleae, which co-localized with hair cell debris (Fig. 7A, B). Using confocal imaging and 3-D reconstruction, we found that nearly all myosin VIIA-immunoreactive material was located above the lumenal surface of the sensory epithelium (Fig. 7C, D). It should be noted that tectorial membranes were not removed from these specimens, so it is likely that hair cell debris was extruded from the epithelium and then held in place by the overlying tectorial membrane. In contrast, very little myosin VIIA-labeled material remained within the sensory epithelium (Fig. 7E, F). Quantification of hair cells within the sensory epithelium revealed that macrophage-depleted cochleae contained 0.4±0.7 hair cells/10,000 µm^2^, while cochleae with normal macrophage populations contained 1.1±2.1 hair cells/10,000 µm^2^. Together, these data suggest that apoptotic hair cells are removed from the lumenal surface of the sensory epithelium and that this process does not require the assistance of resident macrophages.

### Elimination of Macrophages does not Affect Hair Cell Regeneration

**Figure 8:**
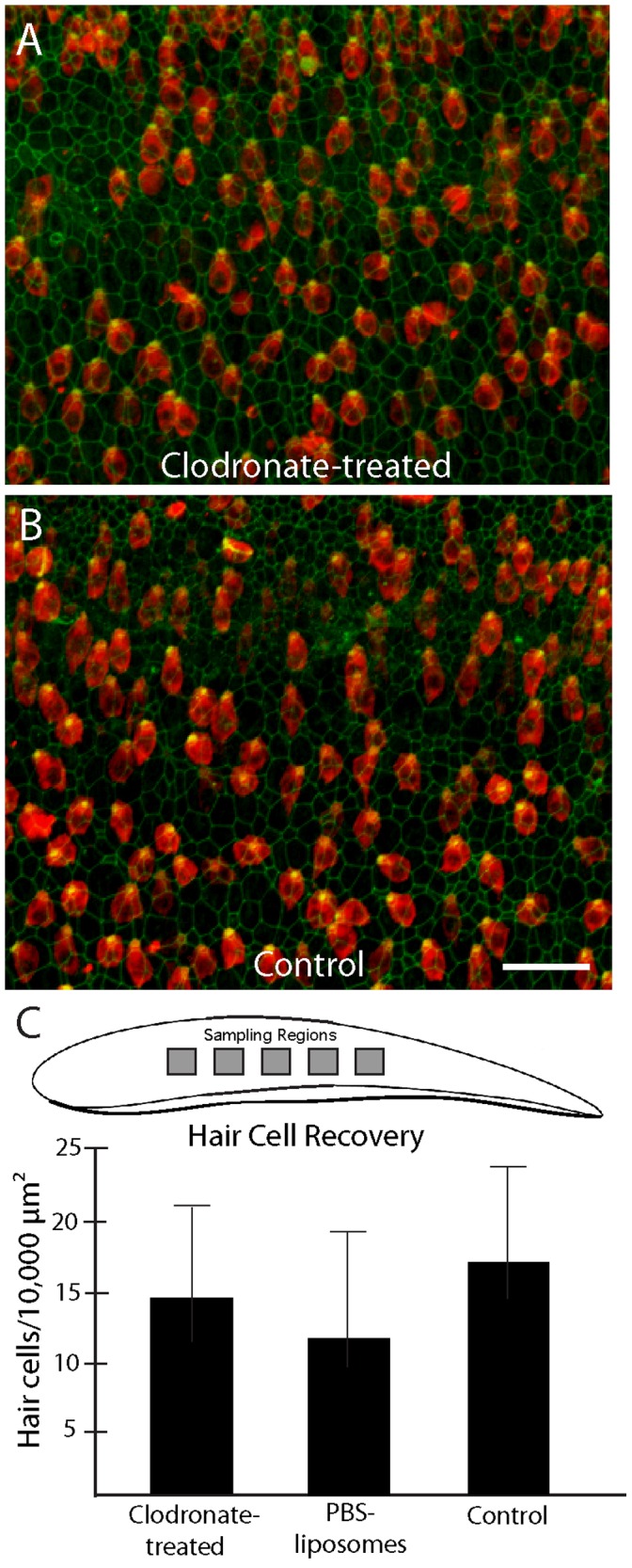
Depletion of cochlear macrophages does not affect hair cell recovery after ototoxic lesion. Cochlea were treated with clodronate-containing or ‘empty’ liposomes, or received no pretreatment. They were then incubated for 24 hr in 1 mM streptomycin (to kill hair cells) and allowed to recover *in vitro* for an additional seven days. Nearly identical levels of hair cell recovery were observed in all specimens, regardless of pretreatment (A, B). Quantification of hair cell density from five 100×100 µm regions/cochlea confirmed that macrophage depletion had no effect on hair cell regeneration (C). Scale bar = 30 µm.

Having established that macrophage depletion did not affect the clearance of hair cell debris after ototoxic injury, we next examined whether macrophages influence the process of hair cell regeneration. Cochleae were incubated for 24 hr in either: (1) clodronate-containing liposomes, (2) ‘empty’ (PBS-containing) liposomes, or (3) normal medium without liposomes. Each treatment group consisted of 10 cochleae. After these treatments, all specimens were thoroughly rinsed with fresh medium, incubated for 24 hr in medium that contained 1 mM streptomycin, and then thoroughly rinsed in fresh medium and maintained in culture for seven days (in order to allow time for hair cell recovery). After fixation, the specimens were immunolabeled for myosin VIIA and the numbers of hair cells were quantified from five 100×100 µm regions in the central region of each specimen (Fig. 8). Regardless of pretreatment, all cochleae contained ∼10–15 hair cells/10,000 µm^2^, which is an approximately15-fold increase over the hair cell density observed at 48 hr after ototoxic injury (compare Fig. 7G vs. Fig. 8C). This result suggests that elimination of macrophages does not affect the initial stages of hair cell regeneration.

### Elimination of Macrophages does not Affect Supporting Cell Proliferation

**Figure 9:**
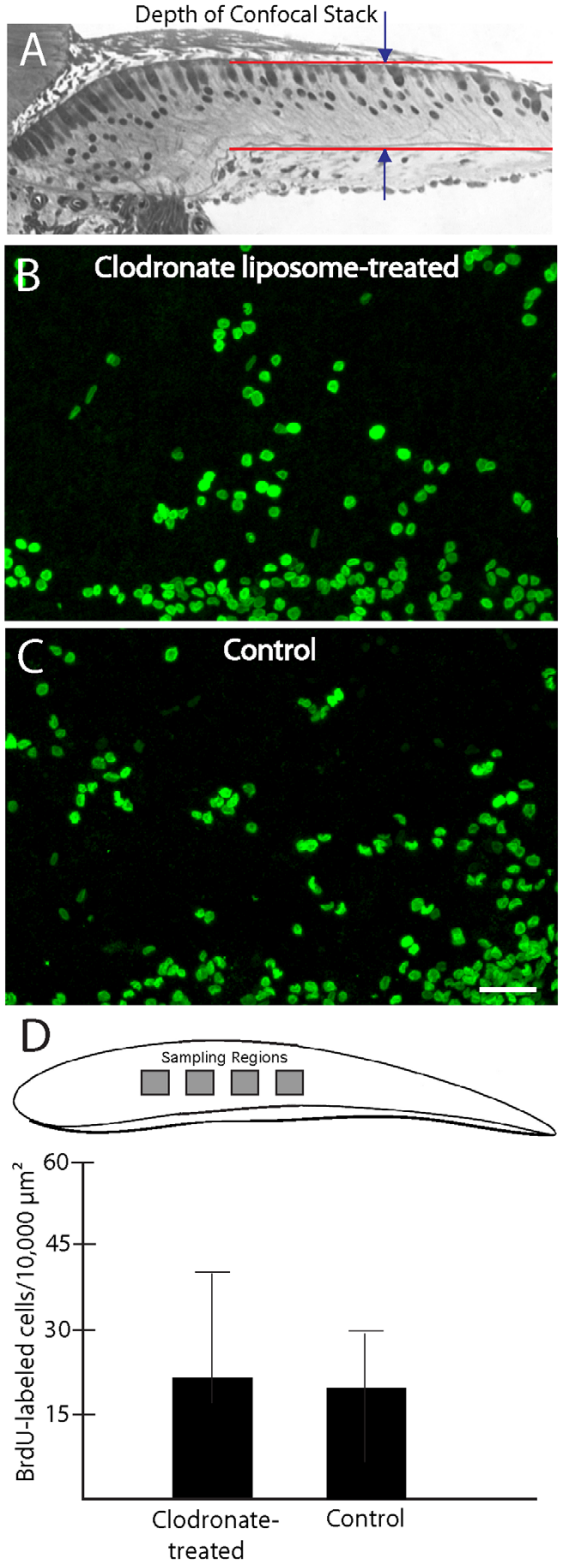
Macrophage depletion does not affect regenerative proliferation. Cochleae were treated for 24 hr. in clodronate-containing or control medium, followed by 24 hr in 1 mM streptomycin. Specimens were then rinsed and maintained for an additional four days in medium that contained BrdU. Confocal microscopy was used to image BrdU-labeled cells within the sensory epithelia of the cochleae (A). Similar patterns of BrdU labeling were observed in clodronate-treated (B) and control (C) cochlea. Quantification of BrdU labeled cells (from four 100×100 µm regions/specimen) confirmed that supporting cell proliferation was not affected by clodronate treatment and macrophage depletion (D, p = 0.28). Scale bar = 30 µm.

The avian cochlea can regenerate hair cells through two distinct mechanisms: (1) the evoked proliferation of supporting cell precursors, or (2) direct transdifferentiation of supporting cells into replacement hair cells [14]. It is possible that macrophage depletion may selectively impact one or the other of these processes, but that normal levels of hair cell replacement may still occur via enhancement of the unaffected mechanism. To resolve this issue, we examined the effects of macrophage depletion on supporting cell proliferation. Cochleae (n = 10) were treated for 24 hr in clodronate-containing liposomes, followed by 24 hr in streptomycin (as described above). Control specimens (n = 10) were run in parallel, but did not receive clodronate. Following streptomycin treatment, all cultures were rinsed and the mitotic tracer BrdU was added to the medium. Specimens were maintained for an additional four days and were then fixed and processed for immunohistochemical labeling of BrdU. Confocal images of the sensory epithelia were obtained and BrdU-labeled cells were quantified from 100×100 µm regions in the central portion of the sensory region. Nearly identical levels of BrdU incorporation were observed in clodronate-treated and control specimens (Fig. 9B, C, D), indicating that macrophage depletion does not alter the regenerative proliferation of cochlear supporting cells.

### Macrophages Depletion Reduces Cell Division within the Basilar Membrane

**Figure 10:**
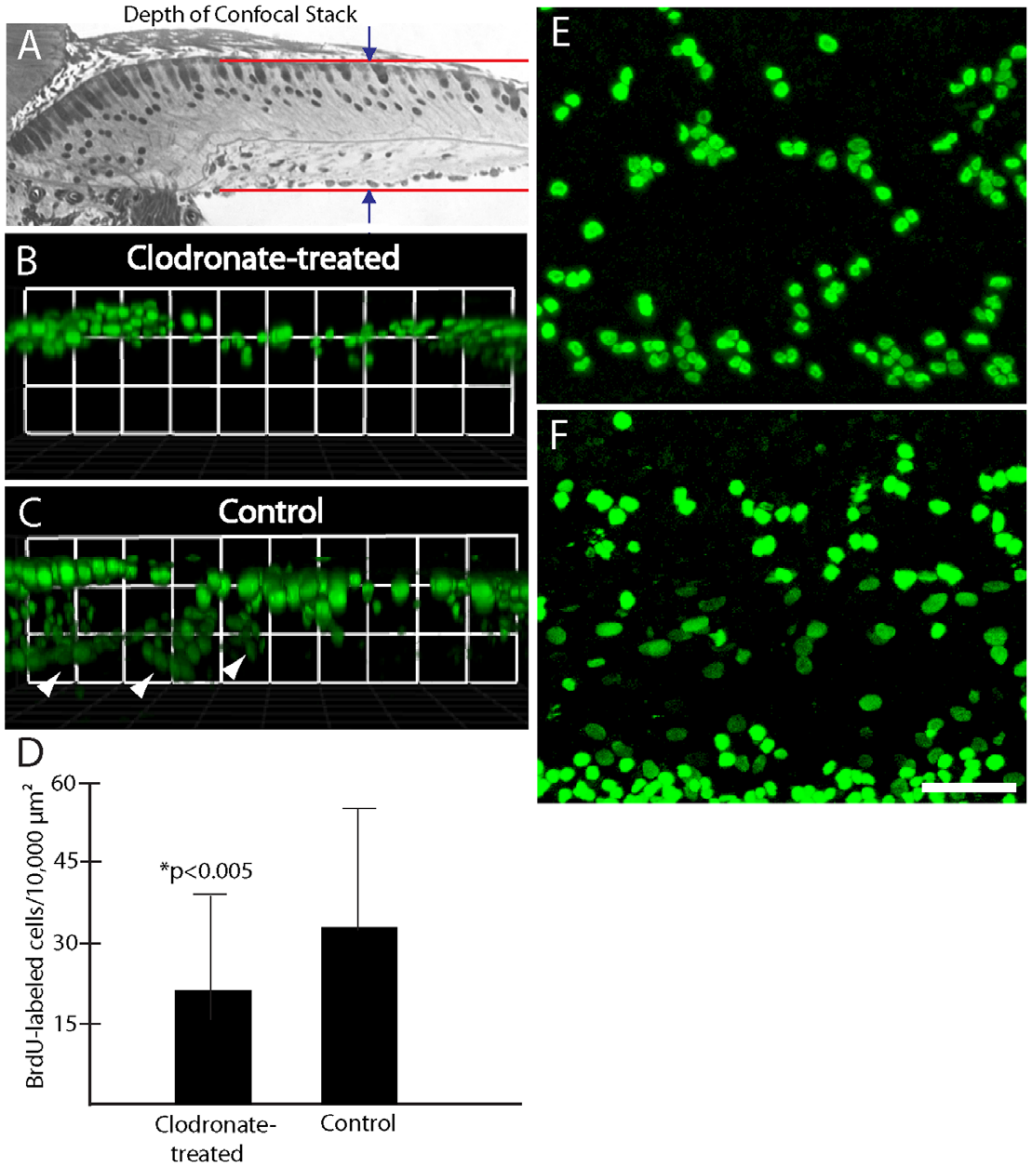
Clodronate treatment reduces proliferation of nonsensory cells in the chick cochlea. (A) Confocal image stacks of BrdU-labeled cells (green) in clodronate-treated and control specimens were obtained by scanning through the entire specimen (including both the sensory epithelium and the underlying basilar membrane region). (B, C) Three-dimensional reconstructions of those images indicated that clodronate treatment selectively reduced proliferation of cells below the sensory epithelium (C, arrowheads). Surface views of clodronate-treated (E) and control (F) cultures also suggested fewer BrdU-labeled cells after clodronate treatment. Quantification of all BrdU-labeled cells in those specimens confirmed that clodronate reduces proliferation (p<0.005). Scale bar = 30 µm.

Although the previous data revealed similar numbers of proliferating supporting cells in clodronate-treated and control cultures (e.g., Fig. 9D), visual inspection of those specimens suggested that macrophage-depleted cochleae contained fewer BrdU-labeled cells than did untreated controls (e.g., Fig. 10E, F). In order to characterize cell proliferation in these specimens more completely, we obtained confocal image stacks through the *entire depth* of the sensory region, which contained both epithelial supporting cells as well as the basilar membrane and associated mesothelial cells (Fig. 10A). Three-dimensional renderings of these confocal stacks revealed that proliferation in clodronate-treated specimens was mainly confined to the sensory epithelium (Fig. 10B), while control specimens also contained numerous proliferating cells in the regions below the sensory epithelium (Fig. 10C). Quantification of all BrdU-labeled cells indicated that clodronate treatment reduced the *total* number of proliferating cells by ∼40% (Fig. 10D). Finally, the morphology of BrdU-labeled nuclei permitted us to tentatively distinguish between epithelial supporting cells and cells located outside the sensory epithelium. Supporting cell nuclei were round and of approximately equal diameter (Fig. 9B, C). Nearly all BrdU-labeled nuclei observed in the clodronate-treated cochleae corresponded to this phenotype (Fig. 10E). However, confocal images of control specimens contained both round nuclei as well as oval-shaped nuclei of various sizes (Fig. 10F). Those nuclei were usually deeper in the tissue and correspond to cells below the basilar membrane.

### Proliferation of Mesothelial Cells and Macrophages in Cochlear Cultures

**Figure 11:**
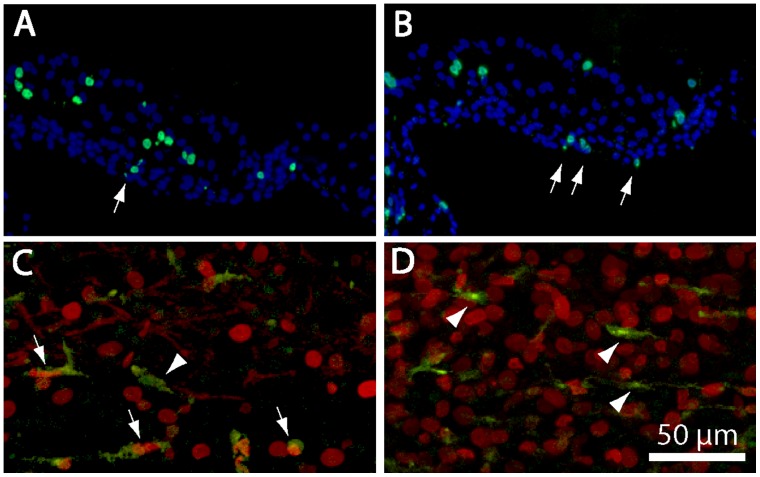
Location and identity of BrdU-labeled cells in cochlear cultures. Frozen sections of cultured cochleae typically contained BrdU-labeled cells (green) in the sensory epithelium and within the basilar membrane (arrows, A, B). Cell nuclei are labeled with DAPI (blue). (C, D) Confocal images of cochleae that were double labeled for KUL01 (green, macrophages) and BrdU (red) indicate that macrophages constituted a minority of proliferating cells (arrows). Numerous quiescent macrophages (arrow heads) were also present. Scale bar (for images C and D) = 50 µm.

Given that the depletion of macrophages reduced proliferation among cells below the basilar membrane, we then focused on identifying the affected cells. A previous study had shown evidence for the proliferation of macrophages in cochlear cultures [4], so it was possible that the observed reduction in proliferation was simply due to the elimination of those cells. In order to resolve this issue, we first compared the numbers of KUL01-labeled macrophages with the numbers of proliferating cells within the cochlear regions shown in Fig. 9D. At one day after streptomycin treatment, cultured cochleae contained 6.4±3.3 macrophages/10,000 µm^2^ while, at four days post-streptomycin, cochleae contained 5.0±2.5 macrophages/10,000 µm^2^ (n = 22/24 samples from six cochleae/time point). However, our BrdU data indicate that clodronate treatment reduced proliferation in these regions by ∼12 cells/10,000 µm^2^ (Fig. 10D). These data suggest that the simple elimination of resident macrophages is not sufficient to account for the clodronate-evoked decrease in proliferation. We also examined the location and morphology of BrdU-labeled cells in frozen sections of cochleae that were fixed at four days after treatment with 1 mM streptomycin. Labeled nuclei within the sensory epithelium (supporting cells) were typically larger and rounder than labeled nuclei below the sensory region (arrows, Fig. 11 A, B). Double-labeling with the KUL01 antibody indicated that both proliferating and quiescent macrophages were present in all cultures (Fig. 11C, D). Notably, however, proliferating macrophages constituted a minority of BrdU-labeled cells within all regions of the cochlea. Proliferating cells below the basilar membrane often had elliptically-shaped nuclei and were situated between the basilar membrane and the fluid space of scala tympani. Such cells are generally classified as mesothelial or tympanic border cells [15].

## Discussion

The present study confirms and extends prior observations, demonstrating that the avian cochlea contains a resident population of macrophages and that increased numbers of macrophages are attracted toward sites of hair cell injury [4], [6], [16]. Most somatic tissues contain resident populations of macrophages, which play a key role in innate immunity and serve as first responders after injury. Our observations indicate that the avian cochlea contains a large population of resting macrophages that are concentrated just outside of the inferior border of the sensory epithelium. Ototoxic injury appears to cause these macrophages to migrate across the inferior border into scala tympani, below the hair cell lesion. However, few macrophages appeared to enter the sensory epithelium, and we observed limited evidence for macrophage phagocytosis of apoptotic hair cells. Moreover, selective elimination of macrophages from cultured cochleae did not affect debris clearance from the injured sensory epithelium and did not impair hair cell regeneration. Instead, macrophage elimination reduced the proliferation of cells beneath the sensory epithelium, including the mesothelial cells that line the basilar membrane. Taken together, our results suggest that macrophages may participate in the maintenance and homeostasis of nonsensory cochlear tissues, but do not serve a critical role in the initiation of hair cell regeneration.

### Phenotypic Identity of Leukocytes in the Avian Ear

Results of prior studies suggest that the avian cochlea contains several distinct classes of leukocytes. Initial studies, based on acid phosphatase histochemistry, revealed numerous resident macrophages in the sensory and nonsensory regions of the chick cochlea [4]. A subsequent study reported that the chick inner ear contained cells that resembled macrophages and that expressed the leukocyte-common antigen CD45 [6]. Use of more specific immunolabeling reagents indicated that the avian ear contains several types of leukocytes, as well as B- and T-lymphocytes [16]. Although the role of these cells in the context of inner ear function remains undetermined, such data challenge the notion that the inner ear is ‘immune-privileged.’ The present study used the KUL01 antibody, which recognizes an unidentified epitope on the extracellular surface of chick macrophages. Prior studies have shown that KUL01 selectively labels chick monocytes, macrophages, and activated (but not resting) microglia [10]. As such, the present results provide the most definitive characterization to-date of macrophages in the avian cochlea, and suggest that macrophages are widely distributed in the nonsensory structures of the cochlea, but are relatively rare in the sensory epithelium.

### Spatial Distribution of Macrophages in the Normal and Injured Cochlea

One novel finding of the present study was that the inferior (abneural) region of the avian cochlea hosts a large population of resting macrophages. This epithelial region is comprised of so-called hyaline and cuboidal cells, which form the inferior boundary of the sensory region and connect the basilar membrane to the surrounding cartilage. Hyaline cells receive extensive efferent innervation [17], which may regulate the mechanical properties of the basilar membrane [18], [19]. The functional significance of this localized collection of macrophages just outside of the sensory region is unclear, but it is conceivable that large numbers of resting macrophages within or below the sensory epithelium might interfere with cochlear mechanics or with other aspects of the transduction process. It is also not clear what chemical signals cause macrophages to remain within the hyaline cell region in the normal cochlea, but then summon them into the basilar membrane after ototoxic injury. Macrophage activation and motility are regulated by a large number of cytokines and chemokines, and little is known about the expression of these molecules within the cochlea. Microglia (the resident macrophages of the CNS) are activated and recruited by the release of ATP from injury sites [20], [21]. Prior studies have demonstrated that ATP is also released from sites of hair cell injury in the cochlea, leading to the generation of a propagating Ca^2+^ wave [22], [23]. Given these data, it is tempting to speculate that similar mechanisms might regulate the behavior of resident macrophages in the inner ear and in the CNS. However, the precise relationship between microglia and cochlear macrophages has not been determined and resolution of this issue awaits further experimentation.

### Macrophages are not Required for Removal of Hair Cell Debris After Ototoxic Injury

One established function of tissue macrophages is to quickly remove the debris of apoptotic cells, in order to preserve epithelial integrity and to prevent an inflammatory response. Although it is reasonable to suspect that cochlear macrophages might contribute to the clearance of debris after hair cell injury, definitive evidence remains lacking. Prior studies of the avian ear have revealed at least two distinct mechanisms of ‘corpse’ removal after hair cell death. In the cochlea, most dying hair cells appear to be directly extruded from the apical surface of the sensory epithelium [2], [24]. Once removed from the epithelium, the fate of cellular debris is unknown. A somewhat different form of clearance is observed in the chick utricle, following aminoglycoside ototoxicity. In that situation, the apical portions of dying hair cells (containing the stereocilia and cuticular plate) are extruded from the lumenal surface, while the basal portions of those cells are phagocytosed by surrounding supporting cells [3]. Although the utricular sensory epithelium does contain some macrophages, those cells do not appear to engulf dying hair cells [3]. Our results are in general agreement with these previous studies. Specifically, we found that the extrusion of apoptotic hair cells did not require the presence of macrophages, and we observed very limited evidence for hair cell phagocytosis by macrophages (e.g., Fig. 4). Together, our data indicate that removal of apoptotic cells is not the primary function of resident cochlear macrophages.

### Elimination of Cochlear Macrophages does not Affect Hair Cell Regeneration

The ears of nonmammalian vertebrates are capable of regenerating hair cells after acoustic trauma or aminoglycoside ototoxicity [25]. Prior studies had suggested that injury-evoked macrophage behavior in lateral line neuromasts of amphibians and in the cochlea and vestibular organs of birds was suggestive of a role in initiating sensory regeneration [4], [5], [6], [26], [27]. Although macrophages have been shown to stimulate tissue repair in other organ systems [28], no prior studies have provided direct evidence for macrophage involvement in hair cell regeneration. The present results suggest that macrophages are not essential for regeneration in the avian cochlea. We observed nearly identical numbers of proliferating supporting cells and regenerated hair cells in normal vs. macrophage-depleted cochleae. Since our studies were conducted *in vitro*, we cannot rule out the possibly that cochlear injury *in vivo* results in the recruitment of additional macrophages from circulation, which may then enhance regeneration. However, regenerative proliferation in the chick cochlea begins within 24 hr of hair cell death [8]. Resident macrophages are likely to be the ‘first responders’ to epithelial injury, and would be best poised to initiate tissue repair and regeneration. Thus, we conclude that macrophages are not necessary for the onset of hair cell regeneration.

### Correlation between Decreased Proliferation and the Loss of Cochlear Macrophages

Although the depletion of cochlear macrophages did not affect the regenerative proliferation of supporting cells, we did find that clodronate treatment reduced cell proliferation below the basilar membrane (Fig. 10B). Some of this reduction may be attributable to the loss of proliferative macrophages. Tissue injury often results in the proliferation of resident macrophages [29], and organotypic cultures of the chick cochlea have been shown to contain some proliferating macrophages [4]. However, our quantitative data suggest that the total numbers of macrophages that reside below the basilar membrane are not sufficient to account for the observed reduction in cell proliferation. As such, it appears that the loss of macrophages leads to decreased division of some other cell type. The basilar membrane of the mature mammalian cochlea is lined by mesothelial cells (also called ‘tympanic border cells’ [30]), and similar cells are present below the basilar membrane in the avian cochlea [15]. Macrophages are known to stimulate proliferation of mesothelial cells in other somatic tissues [31], and it is possible that a similar interaction occurs in the cochlea. The cellular maintenance of the basilar membrane is poorly understood, but is likely to be essential for normal cochlear function. We propose that macrophages may assist in maintaining the basilar membrane, by removing dying mesothelial cells and/or stimulating proliferation among mesothelial cell precursors. Support for this notion is provided by the observations that acoustic injury to the mammalian cochlea leads to both increased numbers of macrophages below the basilar membrane [32] as well as enhanced proliferation of tympanic border cells [33].

### Injury-Evoked Behavior of Cochlear Macrophages: Differences between Birds and Mammals

Finally, our results point to some clear differences in the behavior of both resting and recruited macrophages in the cochleae of birds and mammals. The mouse organ of Corti contains relatively few resident macrophages, but increased numbers of those cells are recruited into the cochlea following acoustic trauma or ototoxic injury [32], [34]. Macrophages in the injured mouse cochlea are particularly evident in the lateral wall, spiral limbus, the spiral ganglion, and along the walls of scala tympani. In contrast, our results indicate that the undamaged avian cochlea contains a relatively large number of resident macrophages and that the initial macrophage response to hair cell injury occurs via the redistribution of resident macrophages rather than recruitment from circulation. It should be emphasized, however, that our *in vivo* experiments only characterized the redistribution of macrophages within 24 hours after ototoxic injury (e.g., Fig. 3), and we cannot rule out the possibility that additional macrophages are recruited into the cochlea at later time points. It is also notable that studies of the injured cochleae of rodents have not observed evidence for macrophage-mediated phagocytosis of hair cell debris [35], [36]. In any case, the present data suggest that the striking differences in regenerative ability observed in the cochleae of birds vs. mammals probably cannot be attributed to macrophage-mediated influences. Instead, the regenerative potential of the vertebrate ear is most closely linked to the ability of inner ear supporting cells to either re-enter the cell cycle or undergo phenotypic conversion after hair cell injury [25].

## References

[1] Rosenblatt J, Raff MC, Cramer LP (2001) An epithelial cell destined for apoptosis signals its neighbors to extrude it by an actin- and myosin-dependent mechanism. Curr Biol 11: 1847–1857. 11728307 10.1016/s0960-9822(01)00587-5

[2] Hirose K, Westrum LE, Cunningham DE, Rubel EW (2004) Electron microscopy of degenerative changes in the chick basilar papilla after gentamicin exposure. J Comp Neurol 470: 164–180. 14750159 10.1002/cne.11046

[3] Bird JE, Daudet N, Warchol ME, Gale JE (2010) Rapid elimination of dying sensory hair cells maintains epithelial integrity in the avian inner ear. J Neurosci 30: 12545–12556. 20844149 10.1523/JNEUROSCI.3042-10.2010 PMC2963157

[4] Warchol ME (1997) Macrophage activity in the avian cochlea: demonstration of a resident population and recruitment to sites of hair cell lesions. J Neurobiol 33: 724–734. 9369147

[5] Warchol ME (1999) Immune cytokines and dexamethasone influence sensory regeneration in the avian vestibular periphery. J Neurocytol 28: 889–900. 10900092 10.1023/a:1007026306730

[6] Bhave SA, Oesterle EC, Coltrera MD (1998) Macrophage and microglia-like cells in the avian inner ear. J Comp Neurol 398: 241–256. 9700569 10.1002/(sici)1096-9861(19980824)398:2<241::aid-cne6>3.0.co;2-0

[7] Stone JS, Leano SG, Baker LP, Rubel EW (1996) Hair cell differentiation in chick cochlear epithelium after aminoglycoside toxicity: in vivo and in vitro observations. J Neurosci 16: 6157–6174. 8815898 10.1523/JNEUROSCI.16-19-06157.1996 PMC6579194

[8] Warchol ME, Corwin JT (1996) Regenerative proliferation in organ cultures of the avian cochlea: identification of the initial progenitors and determination of the latency of the proliferative response. J Neurosci 16: 5466–5477. 8757259 10.1523/JNEUROSCI.16-17-05466.1996 PMC6578879

[9] Zeisberger SM, Odermatt B, Marty C, Zehnder-Fjallman AH, Ballmer-Hofer K, et al (2006) Clodronate-liposome-mediated depletion of tumour-associated macrophages: a new and highly effective antiangiogenic therapy approach. Br J Cancer 95: 272–281. 16832418 10.1038/sj.bjc.6603240 PMC2360657

[10] Mast J, Goddeeris BM, Peeters K, Vandesande F, Berghman LR (1998) Characterization of chicken monocytes, macrophages and interdigitating cells by the monoclonal antibody KUL01. Vet Immunol Immunopathol 27: 343–357. 10.1016/s0165-2427(97)00152-9 9613446

[11] Matsui JI, Ogilvie JM, Warchol ME (2002) Inhibition of caspases prevents ototoxic and ongoing hair cell death. J Neurosci 22: 1218–1227. 11850449 10.1523/JNEUROSCI.22-04-01218.2002 PMC6757575

[12] Shang J, Cafar J, Nehmer R, Stone J (2010) Supporting cell division is not required for regeneration of auditory hair cells after ototoxic injury in vitro. J Assoc Res Otolaryngol 11: 203–222. 20165896 10.1007/s10162-009-0206-7 PMC2862922

[13] Van Rooijen N, Sanders A (1994) Liposome mediated depletion of macrophages: mechanism of action, preparation of liposome and applications. J Immunol Methods 174: 83–93. 8083541 10.1016/0022-1759(94)90012-4

[14] Stone JS, Cotanche DA (2007) Hair cell regeneration in the avian auditory epithelium. Int J Dev Biol 51: 633–647. 17891722 10.1387/ijdb.072408js

[15] Girod DA, Duckert LG, Rubel EW (1989) Possible precursors of regenerated hair cells in the avian cochlea following acoustic trauma. Hearing Res 42: 175–194. 10.1016/0378-5955(89)90143-3 2606802

[16] OHalloran EK, Oesterle EC (2004) Characterization of leukocyte subtypes in chicken inner ear sensory epithelia. J Comp Neurol 475: 340–360. 15221950 10.1002/cne.20162

[17] Zidanic M (2002) Cholinergic innervation of the chick basilar papilla. J Comp Neurol 445: 159–175. 11891660 10.1002/cne.10160

[18] Cotanche DA, Henson MM, Henson OW (1992) Contractile proteins in the hyaline cells of the chicken cochlea. J Comp Neurol 324: 353–364. 1401266 10.1002/cne.903240306

[19] Lippe WR, Zirpel L, Stone JS (2002) Muscarinic receptors modulate intracellular Ca^2+^ concentration in hyaline cells of the chicken basilar papilla. J Comp Physiol A 188: 381–395. 10.1007/s00359-002-0312-z 12073083

[20] Davalos D, Grutzendler J, Yang G, Kim JV, Zuo Y, et al (2005) ATP mediates rapid microglial response to local brain injury *in vivo* . Nature Neurosci 8: 752–758. 15895084 10.1038/nn1472

[21] Sieger D, Moritz C, Ziegenhals T, Prykhozhij S, Peri F (2012) Long-range Ca^2+^ waves transmit brain-damage signals to microglia. Dev Cell 22: 138–148. 10.1016/j.devcel.2012.04.012 22632801

[22] Gale JE, Piazza V, Ciubotaru CD, Mammano F (2004) A mechanism for sensing noise damage in the inner ear. Curr Biol 23: 526–529. 10.1016/j.cub.2004.03.002 15043820

[23] Lahne M, Gale JE (2008) Damage-induced activation of ERK1/2 in cochlear supporting cells is a hair cell death-promoting signal that depends on extracellular ATP and calcium. J Neurosci 28: 4918–4928. 18463245 10.1523/JNEUROSCI.4914-07.2008 PMC6670733

[24] Cotanche DA (1987) Regeneration of hair cell stereociliary bundles in the chick cochlea following severe acoustic trauma. Hearing Res. 30: 181–196. 10.1016/0378-5955(87)90135-3 3680064

[25] Warchol ME (2011) Sensory regeneration in the vertebrate inner ear: differences at the levels of cells and species. Hearing Res 273: 72–79. 10.1016/j.heares.2010.05.004 20488231

[26] Balak KJ, Corwin JT, Jones JE (1990) Regenerated hair cells can originate from supporting cell progeny: evidence from phototoxicity and laser ablation experiments. J Neurosci 10: 2502–2512. 2388077 10.1523/JNEUROSCI.10-08-02502.1990 PMC6570262

[27] Jones JE, Corwin JT (1995) Regeneration of sensory cells after laser ablation in the lateral line system: hair cell lineage and macrophage behavior revealed by time-lapse video microscopy. J Neurosci 16: 649–662. 10.1523/JNEUROSCI.16-02-00649.1996 PMC6578630 8551349

[28] Stefater JA, Ren S, Lang RA, Duffield JS (2011) Metchnikoff’s policemen: macrophages in development, homeostasis and regeneration. Trends Mol Med 17: 743–752. 21890411 10.1016/j.molmed.2011.07.009 PMC3225647

[29] David S, Kroner A (2011) Repertoire of microglial and macrophage responses after spinal cord injury. Nature Rev Neurosci 12: 388–399. 21673720 10.1038/nrn3053

[30] Slepecky NB (1996) Structure of the mammalian cochlea. In: Dallos P, Popper AN, Fay RR editors. The Cochlea. New York: Springer. 44–129.

[31] Mutsaers SE (2002) Mesothelial cells: their structure, function and role in serosal repair. Respirology 7: 171–191. 12153683 10.1046/j.1440-1843.2002.00404.x

[32] Hirose K, Discolo CM, Keasler JR, Ransohoff R (2005) Mononuclear phagocytes migrate into the murine cochlea after acoustic trauma. J Comp Neurol 489: 180–194. 15983998 10.1002/cne.20619

[33] Roberson DW, Rubel EW (1994) Cell division in the gerbil cochlea after acoustic trauma. Am J Otol 15: 28–34. 8109626

[34] Sato E, Shick HE, Ransohoff RM, Hirose K (2010) Expression of fractalkine receptor CX3CR1 on cochlear macrophages influences survival of hair cells following ototoxic injury. J Assoc Res Otolaryngol 11: 223–23. 19936834 10.1007/s10162-009-0198-3 PMC2862920

[35] Abrashkin KA, Izumikawa M, Miyazawa T, Wang CH, Crumling MA, et al (2006) The fate of outer hair cells after acoustic or ototoxic insults. Hearing Res. 218: 20–29. 10.1016/j.heares.2006.04.001 16777363

[36] Taylor RR, Jagger DJ, Forge A (2012) Defining the cellular environment in the organ of Corti following extensive hair cell loss: a basis for future sensory cell replacement in the cochlea. PLoS One 7: e30577. 22299045 10.1371/journal.pone.0030577 PMC3267727

